# Opportunities and Challenges for Surveillance of Congenital Anomalies in Sub‐Saharan Africa

**DOI:** 10.1002/bdr2.70075

**Published:** 2026-06-10

**Authors:** Emma Kalk, Helen Louise Malherbe, Ushma C. Mehta, Daniel Mumpe‐Mwanja, Modiegi Dianah Diseko, Adejumoke Idowu Ayede, Phyllis Kisa, George Bello, Caroline Bonareri Osoro, Lee Fairlie, Audrey Chepkemoi, Hamisi K. Shabani, Ali Sie, Samrawit Abebaw Tegene, Robert Serunjogi, Aminkeng Zawuo Leke, Helen Dolk, Linda Barlow Mosha, the sub‐Saharan African Congenital Anomalies Network (sSCAN)

**Affiliations:** ^1^ Centre for Integrated Data and Epidemiological Research, School of Public Health University of Cape Town Cape Town South Africa; ^2^ Centre for Human Metabolomics, Desmond Tutu School of Medicine, Faculty of Health Sciences North‐West University Potchefstroom South Africa; ^3^ Division of Clinical Pharmacology, Department of Medicine University of Cape Town Cape Town South Africa; ^4^ Makerere University–Johns Hopkins University Research Collaboration (MU‐JHU) Kampala Uganda; ^5^ Botswana Harvard Health Partnership Gaborone Botswana; ^6^ Department of Paediatrics, College of Medicine, University of Ibadan and University College Hospital, Ibadan, Nigeria and Centre for African Newborn Health and Nutrition University College Hospital Ibadan Nigeria; ^7^ Makerere University College of Health Sciences Kampala Uganda; ^8^ International Training and Education Centre Malawi and Ministry of Health, Public Health Institute of Malawi Lilongwe Malawi; ^9^ Population and Health Impact Surveillance Group KEMRI‐Wellcome Trust Research Programme Nairobi Kenya; ^10^ Centre for Global Health Research Kenya Medical Research Institute Kisumu Kenya; ^11^ Wits RHI, Faculty of Health Sciences University of the Witwatersrand Johannesburg South Africa; ^12^ Moi Teaching and Referral Hospital Eldoret Kenya; ^13^ Muhimbili Orthopaedic Institute Dar es Salaam Tanzania; ^14^ Centre de Recherche en Santé de Nouna Nouna Burkina Faso; ^15^ Hararghe Health Research Haramaya University Harar Ethiopia; ^16^ Centre for Maternal and Infant Research Health Research Foundation (HRF) Buea Cameroon; ^17^ School of Medicine Ulster University Belfast UK; ^18^ Syneos Health Morrisville North Carolina USA

**Keywords:** community of practice, congenital anomalies, regional network, sub‐Saharan Africa, surveillance

## Abstract

**Introduction:**

Congenital anomalies (CA) are ranked as the 3rd and 4th leading cause of neonatal and under‐5 mortality, respectively. Over 30% of infants born with a serious CA are found in sub‐Saharan Africa. Yet there are few robust epidemiological data on the burden and distribution of CA and associated mortality, disability, and morbidity, which limits research and investment in clinical and public health interventions. The sub‐Saharan African Congenital Anomalies Network (sSCAN) was established in 2021 to address the fragmented research, surveillance, and care programmes for children with CA in the region.

**Methods:**

The sSCAN established a community of practice with the long‐term aim to promote the prevention and early diagnosis of CA and care for children and families, determining prevalence, promoting preventive interventions, and improving health outcomes by: (1) Sharing resources and multidisciplinary expertise; (2) Establishing a common response to public health questions; (3) Pooling and comparing data between countries; (4) Capacity building.

**Results:**

The sSCAN includes 16 projects across 12 countries, in addition to other stakeholders in sub‐Saharan Africa. Most projects are fully or jointly supported by donor partners with a specific disease/exposure focus: collaboration within Africa is uncommon. Lack of data harmonization, varied funding streams and related data access, and country‐ and institution‐specific data‐protection legislation limited regional data sharing. The sSCAN has hosted 15 webinars which are available with other resources on the website, and supported grant applications and publications.

**Conclusion:**

The sSCAN platform can provide opportunities to build capacity, share expertise, and optimize data use toward contextually‐relevant solutions for CA in sub‐Saharan Africa.

## 1 Introduction

There are insufficient data on congenital anomalies (CA) (both structural and functional (Malherbe et al. 2023)) in sub‐Saharan Africa (SSA) to inform health policy and support appropriate health service responses. SSA is unique in its patterns of antenatal exposures and quality and distribution of health care resources. The epidemiological transition in the region is non‐classical characterized by an on‐going burden of infectious diseases, a rising prevalence of non‐communicable diseases, and persistent high birth rates. CA are an important but under‐prioritized contributor to neonatal, infant, and under‐5 morbidity and mortality (Global Burden of Disease Under‐5 Mortality Collaborators 2021), globally responsible for 23.9% of neonatal deaths, 9.4% of deaths in children under 5 in 2019 (Perin et al. 2023); and 7.4% of stillbirths (Lawn et al. 2016). Over 94% of CA are found in low‐ and middle‐income countries (LMIC) where over 95% of related deaths occur (Perin et al. 2023); SSA accounts for 30% of annual global CA (Christianson et al. 2006) but accurate, contemporary data are lacking which undermines resource allocation and limits the ability to detect signals from potentially teratogenic pregnancy exposures. As progress is made addressing perinatal and infectious causes (e.g., HIV, measles, and malaria), the proportion of morbidity and mortality due to CA is increasing, undermining the attainment of United Nations Sustainable Development Goal (SDG)‐3 in SSA (Strong et al. 2024). Affected children often remain undiagnosed or are misdiagnosed due to healthcare providers' lack of knowledge of CA and limited laboratory services or referred late for specialist care due to stigma and misbeliefs and inadequate referral pathways resulting in irreversible progression of the condition. Surviving children live with severe lifelong disability, placing families in financial hardship with little respite care or mental health support.

The burden of CA in SSA results from the combination of a high prevalence of risk factors (e.g., poverty‐related and dietary exposures, infectious diseases, medicine use [including traditional medicines], and environmental toxins), and a lack of healthcare resources and infrastructure for diagnosis and treatment. Many CA are preventable through public health interventions, particularly those caused by teratogens and dietary deficiencies. There are very few experts in SSA for the diagnosis and treatment (including rehabilitation) of children with CA.

Priority research appropriate to the African context is needed to guide preventive and healthcare initiatives to tackle the high burden and impact of CA in SSA. However, existing research programmes in the region are fragmented and often depend on collaborations with high‐income countries and not between SSA countries.

In Europe and South America, regional CA networks have been highly successful in promoting research and collaboration (i.e., the EUROmediCAT & EUROlinkCAT research projects that arose from the EUROCAT network (Weatherall et al. 1984); and the Latin American Network for Congenital Malformation Surveillance—ReLAMC, established in response to the Zika virus epidemic in Latin America (Orioli et al. 2020)).

SSA was the only region without a CA network (Cardoso‐Dos‐Santos et al. 2020). An opportunity was presented to address this gap in the form of a UK Research and Innovation Medical Research Council (UKRI MRC) call for seed funding to support pan‐African collaborations. The application was successful in the context of the historical lack of resources and research, the Zika virus epidemic in Latin America (Duarte et al. 2017), concerns about antenatal HIV‐integrase inhibitor (dolutegravir) exposure and neural tube defects in Botswana (Zash et al. 2019), and the then current capacity‐building initiatives for CA surveillance by the World Health Organization (WHO), International Clearing House for Birth Defects Surveillance Research (ICBDSR), and US Centers for Disease Control (CDC).

## 2 Methods

The sub‐Saharan African Congenital Anomalies Network (sSCAN) was established in 2021, supported by a 12‐month UKRI MRC seed grant and modeled on the above collaborations. Initial co‐applicants included academic clinicians and epidemiologists working in CA research in SSA who were drawn from personal contacts and by reaching out to published authors in the field. Researchers from Botswana, Cameroon, Ghana, Kenya, Malawi, Nigeria, South Africa, Tanzania, and Uganda were represented on the original application. A snowball approach with networking at regional and international meetings and conferences, and outreach to patient advocacy and support groups has grown the organization. General membership is drawn from attendees of the sSCAN webinars (with their permission) and via a link and email address available to interested parties on the sSCAN website. Relevant publications, conferences, webinars, and funding calls are circulated via the mailing list. We continue to encourage participation, especially from West and Central Africa.

The sSCAN comprises a multidisciplinary partnership of researchers, clinicians, epidemiologists, regulators, government representatives, and patient advocates from across SSA and has the support of the WHO, EUROCAT, ReLAMC, and the ICBDSR. These stakeholders have been engaged since the launch and contribute training and resources via the sSCAN website and participation in regular webinars. The Network includes existing pregnancy and CA surveillance research projects at various stages of implementation led by collaborators, clinicians engaged in the care and treatment of children affected by CA, and patient support and advocacy organizations. There is no data repository at present.

The initial UKRI MRC grant specified funding for website development via The Global Health Network (TGHN) (https://tghn.org/), the first in‐person the sSCAN General Assembly in Kampala, Uganda (2022) and limited administrative support at the fund‐holders' institutions. This was spread over 2 years owing to the disruptions of the COVID‐19 pandemic. Subsequent support from the Bill and Melinda Gates Foundation (BMGF) via the Ubomi Buhle Pregnancy Exposure Registry (PER) in South Africa (Mehta et al. 2025) extended website maintenance and the sSCAN webinars to 2025 and sponsored the second in‐person the sSCAN General Assembly in Cape Town, South Africa (2023). The attendees of both General Assemblies were fully funded by the sSCAN. There is no salary support for Steering Committee members or administrators. Considerable uncompensated time has been required by the sSCAN leadership to maintain the website, organize webinars, represent the Network at regional and international meetings, and organize the General Assemblies. Efforts have been made to mentor less experienced colleagues, engaging them in these activities.

At the first and second General Assemblies, the sSCAN constitution was developed and ratified, and the leadership roles formalized. The constitution (Supporting Information), meeting reports, and minutes of Steering Committee meetings are available on the Governance pages of the website (https://sscan.tghn.org/).

The sSCAN has adopted a Triple Surveillance Approach, including both CA and rare diseases within its remit and expanding the concept of surveillance to include prevalence of conditions, of known causes and of health outcomes (Botto and Mastroiacovo 2018). The mission statement of the Network focuses on important drivers of CA‐related neonatal and under‐5 morbidity and mortality in SSA and includes the following: (1) surveillance (including identification and prevention of risk factors); (2) improving survival and reducing disability in affected children (including diagnosis, medical and surgical treatment, and rehabilitation); and (3) engagement of affected communities in developing the research agenda. Research activities in these areas remain isolated and uncoordinated.

The overall approach has been to provide a platform to share resources and multidisciplinary expertise (this included capacity building and support for surveillance centers, to establish a common response to public health questions and to pool and compare data between countries). The specific aims of the initial seed project were operationalized in three work packages: (1) to establish and build the sSCAN, establish methods for data sharing, establish a website, and agree on priorities for research subgroups; (2) to develop a position paper on the burden of birth defects in SSA in order to make an appropriate case for national funding of public health actions and treatment services; and (3) to scope potential harnessing of new technologies for congenital anomaly surveillance, prevention and care. Cross‐cutting themes included capacity building, collaborative activities, and the submission of joint funding research proposals.

No research involving human subjects is presented in this article and no ethical review was required. The individual projects affiliated with the sSCAN all have Institutional Review Board approvals from the relevant institutions.

## 3 Results

The objectives of the work packages 1 and 2 have been achieved, in part or in full. The sSCAN was launched and is maintained; an electronic footprint has been established, and collaborative academic outputs generated.

After the conclusion of the initial funding, the Directorship moved to Cape Town, South Africa. The Steering Committee meets 6‐weekly and is engaged with planning webinars on the TGHN platform, funding applications, and organizing the second sSCAN General Assembly at which the governance documents were ratified.

### 3.1 Membership

The sSCAN has grown organically from the original co‐investigators to include 16 CA surveillance studies/projects at place or in development, representing 12 countries in SSA (Table 1).

**TABLE 1:** Surveillance and research projects affiliated with the sSCAN.

Project	Organizations and funders	Population	Congenital anomalies	Focus	Country/ies
Toward an Africa platform for congenital anomalies & birth defects surveillance in sub‐Saharan Africa	Centre De Recherche En Sante De Nouna African Academy of Science Grand Challenges Africa	Population‐based	Selected external anomalies visible on surface examination	Infectious exposures	Burkina Faso & Cote d'Ivoire
Cameroon Registry for Congenital Anomaly Surveillance	Health Research Foundation Ministry of Health	Hospital‐based	Selected external anomalies visible on surface examination	Traditional medicine exposures	Buea, Cameroon
Child Health & Mortality Prevention Surveillance (CHAMPS) (Bassat et al. 2023)	Bill & Melinda Gates Foundation	Population‐based (sentinel sites)	All major malformations	Mortality surveillance	Ethiopia, Kenya, Mali, Mozambique, Nigeria, Siera Leone, South Africa (Bangladesh, Pakistan)
Spina bifida and Anencephaly in Ethiopia: folate for prevention (SALT) (Abebaw et al. 2025)	Bill & Melinda Gates Foundation	Population‐based (Health and Demographic Surveillance Systems sites)	Neural tube defects	Neural tube defect surveillance and follow‐up	Harar, Ethiopia Kersa, Ethiopia
MANGO Study (Humphrey et al. 2025; Shafi et al. 2024)	Moi University, Kenya NIAID	Hospital‐based	All major malformations	Antiretroviral therapy (ART exposures)	Eldoret, Kenya
Malarian in Mothers and Babies (MiMBa) Pregnancy Registry	Kenya Medical Research Institute Medicines for Malaria Venture London School of Hygiene & Tropical Medicine	Population‐based (district)	All external anomalies visible on surface examination and congenital heart disease	Anti‐malarian exposures especially first trimester	Kisumu, Kenya (Burkina Faso)
The Malawi Birth Defects Surveillance Project	Ministry of Health US CDC	Hospital‐based	Selected external anomalies visible on surface examination	Antiretroviral therapy (ART exposures)	Malawi
National Birth Defects Surveillance, Nigeria	Ministry of Health	Hospital‐based	Selected external anomalies visible on surface examination		Nigeria
Aminu Kano Teaching Hospital Registry for Congenital Anomalies	Aminu Kano Teaching Hospital Bayero University, Kano	Hospital‐based	All major malformations		Kano, Nigeria
Tsepamo Study (Zash et al. 2019, 2018)	Botswana‐Harvard AIDS Institute Partnership NIH	Hospital‐based (75% of population)	Selected external anomalies visible on surface examination	Antiretroviral therapy (ART exposures)	Botswana
Ubomi Buhle Pregnancy Exposure Registry (Mehta et al. 2025)	University of Cape Town (Shafi et al. 2024; Kalk et al. 2024, 2022) Wits RHI Wits VIDA Research Unit Raheema Moosa Mother and & Child Hospital Health Systems Trust Provincial Departments of Health US CDC Bill & Melinda Gates Foundation	Population‐based (sentinel sites)	Selected external anomalies visible on surface examination	Antiretroviral therapy (ART exposures)	South Africa
Hospital‐based birth defects surveillance in Uganda (Barlow‐Mosha et al. 2022; Kalibbala et al. 2022; Serunjogi et al. 2025)	Makerere University‐Johns Hopkins University research Collaboration & US CDC	Hospital‐based	Selected external anomalies visible on surface examination	Antiretroviral therapy (ART exposures)	Kampala, Uganda
Assessment of fetal anomaly scans in Uganda	Makerere University College of Health Sciences	Hospital‐based	All major malformations	Fetal ultrasound diagnosis and referral	Kampala, Uganda
Birth Outcome Surveillance, after Dolutegravir, eSwathini (Gill et al. 2023)	Ministry of Health, eSwathini ViiV Healthcare through EGPAF‐ASPIRE project	Hospital‐based	Selected external anomalies visible on surface examination	Antiretroviral therapy (ART exposures)	eSwathini
Tanzania Birth Defects Surveillance (Mangat et al. 2018)	Ministry of Health US CDC	Population‐based (district)	Selected external anomalies visible on surface examination	Antiretroviral therapy (ART exposures)	Tanzania
Planned: BICON	Busitema University NEUROKIDS	Population‐based (district)	Selected external anomalies visible on surface examination		Busia, Uganda

Abbreviations: EGPAF‐ASPIRE, Elizabeth Glaser Pediatric AIDS Foundation Attain & Sustain 95‐95‐95, Prevent New Infections and Reach All Populations for Epidemic Control Activity; NIAID, National Institute of Allergy & Infectious Diseases; NIH, National Institutes of Health; US CDC, United States Centers for Disease Control; VIDA, Vaccines and Infectious Diseases Analytic Research Unit.

Almost all projects are supported, fully or in part, by global partners or research organizations and have an exposure‐specific focus (e.g., antiretroviral therapy [ART] for the treatment of HIV in pregnancy; anti‐malarial therapy; antenatal Zika virus exposure). In this respect the research and related service agendas are partner‐driven. Each project has specific guidelines for standard case definitions, data collection protocols and reporting criteria with no harmonization between projects. The acute changes in US federal funding policy and subsequent impact on the funding landscape in 2025 have significantly undermined CA surveillance and services in SSA. The US CDC‐supported initiatives in Uganda and Malawi closed in 2025. The Ubomi Buhle PER in South Africa, jointly supported by the US CDC and the Gates Foundation will conclude in late 2026 as funding is not renewed. The MANGO study in Kenya has ceased enrolment and the Tsepamo study in Botswana has curtailed activities (both have been supported by the US National Institutes for Health [NIH]). The grant support for the MiMBA study in Kenya is also concluding.

Two fully‐sponsored General Assemblies have been held, in 2022 in Kampala, Uganda and in 2023 in Cape Town, South Africa; the third (unsponsored) was held in Johannesburg, South Africa in November 2025 as part of the ICBDSR Annual Meeting programme (https://icbdsr2025.co.za/). The latter two meetings included a free data management pre‐conference workshop conducted by the ICBDSR. In‐person meetings have been extremely impactful in terms of consolidating partnerships and support for the organizational structures of the sSCAN.

### 3.2 Outputs

**FIGURE 1:**
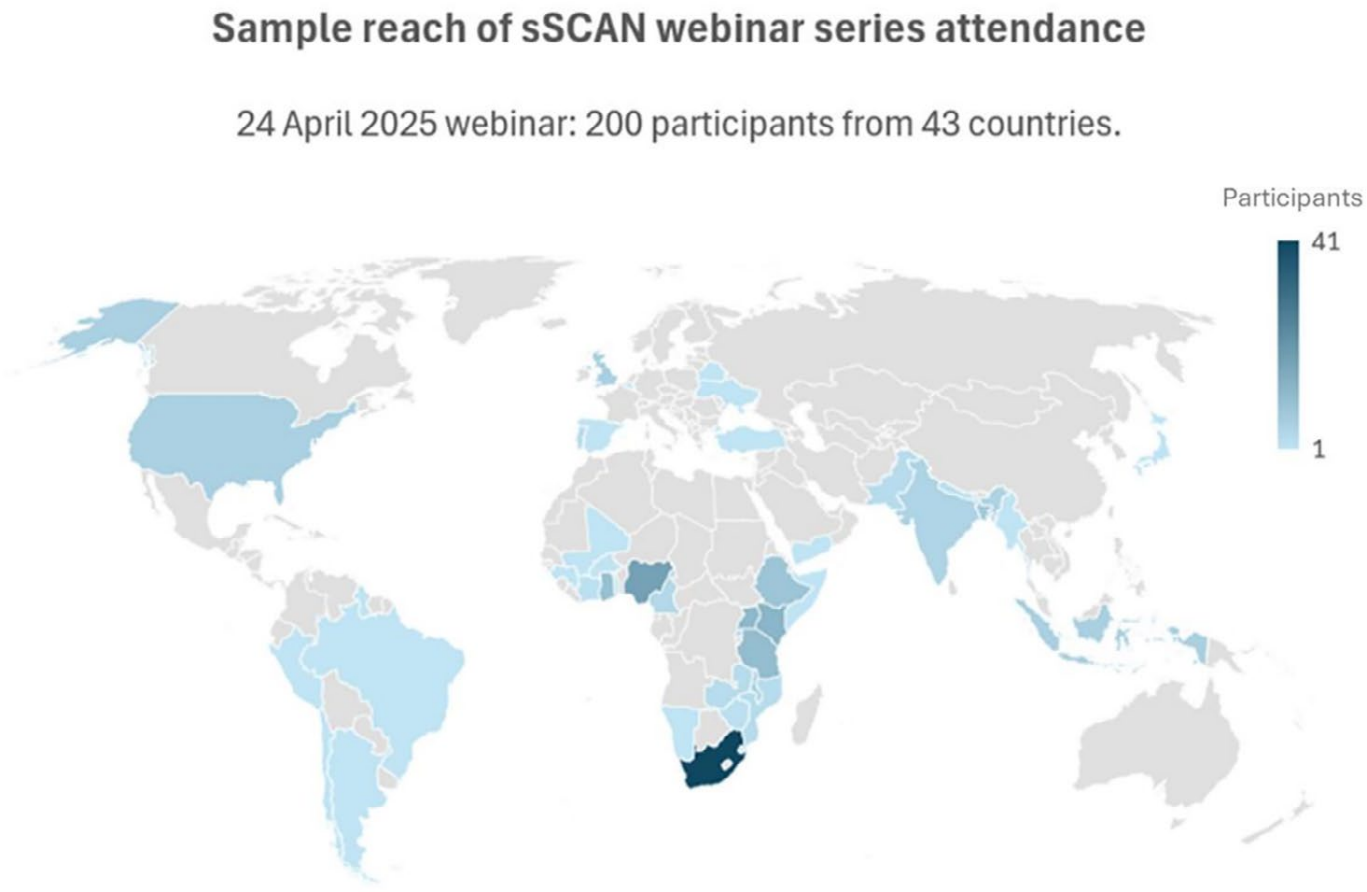
The geographic distribution of attendees of sSCAN webinars (data from 24 April 2025) (The Global Health Network).

The sSCAN has produced a webinar series of 15 broadcasts by African and other scientists from LMIC co‐ordinated by TGHN (Figure 1; Table 2). All are freely available together with other resources on the website. Since April 2025, owing to lack of funding, the sSCAN has been unable to host webinars on the TGHN platform; we have explored alternative arrangements and have co‐hosted a World Birth Defects Day webinar (03 March 2026) with the South African National Department of Health on their Knowledgehub platform.

**TABLE 2:** Scans webinar series (available at https://sscan.tghn.org/webinars/).

Title	Date	Registered	Attended
sSCAN: Birth Defects Surveillance: why and how, the African Experience Part 1	30 June 2021	96	67
Addressing Congenital Anomalies and Triple Surveillance on the Path to meet SDG3 Targets	28 July 2021	73	34
sSCAN: Birth Defects Surveillance: why and how, the African Experience Part 2	25 Aug 2021	72	37
The use of New Technologies for congenital anomaly surveillance, diagnosis and care	29 Sept 2021	55	27
Specialist service provision: pediatric surgery	27 Oct 2021	113	34
Teratogens and Pharmacovigilance	24 Nov 2021	204	53
Neural Tube Defects in sub‐Saharan Africa	12 Oct 2022	175	98
Community Screening Strategies to Improve Equitable Access in Diagnosis and Management of Birth Defects: Lessons from Low and Middle‐Income Countries	11 May 2023	60	24
Congenital Heart Defects in Sub‐Saharan African Countries	17 July 2023	111	63
Advocacy for congenital disorders and rare diseases in sub‐Saharan Africa: an expert discussion	31 Aug 2023	44	34
Childhood Disability in sub‐Saharan Africa	2 Nov 2023	368	129
Orofacial Clefts in sub‐Saharan Africa: Epidemiology, Care and Prevention	30 May 2024	483	150
Congenital Infections associated with Congenital Anomalies in sub‐Saharan Africa	25 July 2024	298	128
Overcoming Barriers to Birth Defect Diagnosis and Management in sub‐Saharan Africa	28 Nov 2024	331	161
Universal newborn screening in low‐ and middle‐income countries	24 April 2025	500	200

Abbreviations: SDG, Sustainable Development Goal; sSCAN, sub‐Saharan African Congenital Anomalies Network.

A collaborative scoping review was conducted to determine the range and scope of published CA studies in SSA and demonstrated that a small number of countries account for the majority of research outputs, which is more likely a reflection of access to resources and expertise than the actual burden of disease. Most studies were conducted at a single hospital site and considered a single type of CA. There was a lack of research on risk factors and prevention, and none assessing the numbers of children without any access to hospital care (Leke et al. 2023).

In addition to scientific peer‐reviewed abstracts by our members, The sSCAN was presented at the International Conference on Birth Defects & Disabilities in the Developing World, 01–04 March 2023, Santiago, Chile and the Third Biennial Rare Diseases Conference (RARE‐X 2024) 14–17 February 2024, Johannesburg South Africa; and invited to address at the 50th Annual Meeting of the ICBDSR, 02–04 September 2024, Prague, Czech Republic. The Network co‐hosted the 51st Annual Meeting of the ICBDSR with the Centre for Human Metabolomics, North–West University, South Africa, 02–05 November 2025, in Johannesburg, South Africa (https://www.icbdsr.org/wp‐content/uploads/2025/11/Abstract‐booklet‐Magaliesburg‐2025_final.pdf.) We secured competitive funding from the South African Medical Research Council to support attendance by delegates from SSA. The sSCAN has applied for competitive research funding as a Network and supported grant applications by affiliates with resources and expertise.

### 3.3 Capacity Building

Resources and expertise supporting the diagnosis, treatment and care for children and families affected by CA are scarce and concentrated in the urban centres of a few countries in SSA. Capacity building has taken multiple forms. Recordings of the webinars, publications and other resources are freely available on the sSCAN website. Less experienced colleagues have been supported in organizing webinars and chairing meetings and with conference submissions and oral presentations. The second and third General Assemblies were preceded by full‐day workshops facilitated by the ICBDSR (30–35 attendees/workshop). Based on the WHO/CDC/ICBDSR birth defect toolkit (WHO 2020), content included practical training in diagnosing and reporting birth defects, with a focus on data quality (completeness and accuracy), coding, and timeliness.

The sSCAN has engaged members in grant applications for the network and supported the individual applications of others.

### 3.4 Data Sharing

A cross‐cutting approach within the sSCAN is data harmonization for data sharing across established research groups to address questions that may require a larger sample size or wider geographical variation. A data‐sharing platform with common definitions and rules will allow consolidation of existing data across the region and allow an accurate and ongoing system to assess the birth prevalence and range of CA in SSA as well as risk factors which may be amenable to prevention.

As the first step, a catalog of data variables has been compiled. However, establishing a common data platform or repository has proved challenging, owing to (1) varied data standards employed by each project; (2) different funding streams, and related ‘ownership’ of and access to data; and (3) country‐ and institution‐specific data protection legislation. Funding constraints have limited progress in this regard. Smaller, condition‐specific federated analyses may present a solution in the short term. As will the definition of a minimum core data set, although this will differ depending on the aims of the individual project, that is, to determine prevalence of a condition, isolated or part of a syndrome; to assess potential teratogenicity of an exposure.

### 3.5 Funding

Since 2023, the sSCAN has submitted applications to large, competitive research funding calls. These efforts to support the organization as a whole have not been successful and our alternative approach is to include budget for the sSCAN website and webinars in other CA‐related applications as well as responding to defined, specific calls, for example, support for conference travel.

Some groups (in Kenya, Malawi, South Africa, and Uganda) affected by the funding constraints have been able to engage with their respective Ministries of Health but with little success.

## 4 Discussion

The establishment of the sSCAN network, notwithstanding the limited funding, has been an important step toward bringing together partners across SSA with a common interest in CA, who operate under similar regional conditions and find strength in collaboration.

In a region of high CA burden, we lack data on the birth prevalence and scope of CA which undermines resource allocation, the development of relevant child nutrition and health policies, and limits the ability to detect signals from potentially teratogenic pregnancy exposures. Health and surveillance services are fragmented and under‐resourced. The sSCAN brings together CA subject experts (clinicians, epidemiologists, patient and family advocates, regulators, and policy makers) in a community of practice to educate, collaborate and share resources to improve diagnosis, identify and mitigate the causes and strengthen access to care for individuals affected by CA in SSA.

The process of organizing and advertising the webinars has been key to expanding membership. Attendance of the webinars has progressively increased, notably after the calls were advertised in TGHN newsletter. In addition, as the Network was formally presented at local (MUH‐JU meeting) and international (RARE‐X, ICBD, and ICBSDR) conferences, interest has been maintained.

As noted, support for affiliated surveillance programmes is external, from donors and partners, and not from national ministries/departments of health. This has meant that priorities are donor‐driven and often focused on a specific condition and/or exposure, for example, HIV and ART, malaria and antimalarial medication. It can be challenging to ensure the alignment of needs of both the donor and recipient; however, the platforms created to monitor specific conditions can be applied across multiple exposures. The vulnerability of depending on external support (as opposed to budget from national health departments) has come into focus with the recent changes in US federal subsidies and subsequent defunding of well‐established and impactful services.

Operational support for the sSCAN was originally generated via research grants. Surveillance is often viewed as a function of government and it has proved difficult to secure funding from research programmes, especially for the Network as a whole. Our subsequent approach has been to apply for components of the sSCAN (website, webinars, and travel) with Steering Committee member and administrative time remaining unfunded. In the current climate, maintaining the electronic footprint of the website and email contact has been prioritized.

Data harmonization and the establishment of a sSCAN shared data platform has been identified as an important step toward establishing collaboration and supporting regional analyses. Despite extensive discussion at the in‐person meetings, this has proved challenging. Data sharing would require approval of the organizations (including Ministries of Health) which secure and are responsible for the data. Institutional and government obligations with respect to participant privacy and consent, as well as national legal frameworks complicate the issue. A solution may be an approach to federated analyses using a common data model or applying for permissions for specific projects. The sSCAN has already compiled a dictionary of the data variables across affiliated projects.

Common themes have emerged around the challenges across the surveillance projects. Completeness of surface examinations of stillborn infants remains a limitation that needs to be addressed through training and support of clinical staff at sentinel surveillance sites; inadequate diagnostic capacity has confined CA surveillance largely to surveillance of major malformations detectable at birth through a simple surface examination (Holmes et al. 2021). Therefore, while considered more common, surveillance of congenital heart defects, other internal anomalies, and anomalies that manifest beyond birth such as metabolic conditions are poorly understood. Coding and classification of CAs is a challenging exercise in the absence of appropriate expertise, and many surveillance projects have resorted to remote diagnosis of conditions by clinical geneticists using photo and video images of cases. Sharing and discussing such challenges across the Network will contextualize the data obtained from individual projects to support pooled analyses while providing opportunities for members to discuss approaches to addressing these challenges.

## 5 Conclusions

The sSCAN successfully established a much‐needed community of practice to address clinical, surveillance, and advocacy needs related to CA in SSA. The Network provides a centralized repository for resources in a fragmented and resource‐constrained setting. As we work toward a more formal common data system to support regional analyses, we continue to expand and sustain clinical care, research, and advocacy for children and families affected by CA in SSA.

## Author Contributions

All authors are founding and or active members of the sub‐Saharan African Congenital Anomalies Network (sSCAN). All were involved in planning the manuscript at the second sSCAN General Meeting. E.K. and H.M. wrote the manuscript which all authors reviewed and approved.

## Funding

UKRI MRC seed grant [MR/T039132/1] (PI Dr. Barlow‐Mosha) and the Bill and Melinda Gates Foundation (BMGF) through the Ubomi Buhle grant INV‐004508.

## Ethics Statement

The authors have nothing to report.

## Consent

The authors have nothing to report.

## Conflicts of Interest

The authors declare no conflicts of interest.

## Supporting information


**Figure S1:** The sSCAN Organogram.

## Data Availability

The data that support the findings of this study are available from the corresponding author upon reasonable request.
