# Supplementary material for: Opportunities and Challenges for Surveillance of Congenital Anomalies in Sub‐Saharan Africa
Source: Birth Defects Res. 2026 Jun 10;118(6):e70075. doi: 10.1002/bdr2.70075 (PMC13254227; doi:10.1002/bdr2.70075)
Supplement: Supplementary file 1 — Figure S1: The sSCAN Organogram. [file BDR2-118-e70075-s001.pdf]

Constitution of the sub-Saharan African Congenital Anomalies Network (sSCAN) (Version 1.0 October 2023)

Background

The sub-Saharan African Congenital Anomalies network (sSCAN) was established in 2020/2021 to address the limited and fragmented surveillance, research and care programmes in the field of congenital anomalies in the region.

There are insufficient data on congenital anomalies in sub-Saharan Africa to inform health policy and support health service responses. Sub-Saharan Africa is unique in terms of patterns of antenatal exposures and quality and distribution of health care resources. The epidemiological transition in the region is not classical with an on-going burden of infectious diseases, a rising prevalence of non-communicable diseases, and persistent high birth rates. Congenital anomalies are an important but under-prioritized contributor to neonatal and under-5 mortality and morbidity. As progress is made perinatal and newborn care and addressing infectious causes (e.g., HIV, measles, malaria), the proportion of mortality and morbidity due to congenital anomalies is increasing. Sub-Saharan Africa accounts for 30% of annual global congenital anomalies Affected children often remain undiagnosed due to healthcare providers' lack of knowledge of congenital anomalies or referred late for specialist care due to inadequate referral pathways contributing to avoidable death and severe lifelong disability, placing families in financial hardship.

The high burden of congenital anomalies in sub-Saharan Africa results from the combination of a high prevalence of risk factors (e.g., poverty, environmental toxins, dietary factors, infectious diseases, medicine use), and a lack of healthcare resources for diagnosis and treatment. Many are preventable through public health interventions.

sSCAN brings together a diverse team of experts and engaged individuals from across multiple disciplines throughout the region providing a platform for existing congenital anomaly research and advocacy activities across sub-Saharan Africa to strengthen research capacity and provide robust evidence-based solutions to the region-specific challenges.

Aims and Objectives

The overall aim of the network is to improve the diagnosis of structural congenital anomalies, promote the identification and prevention of the causes of congenital anomalies and strengthen access to care and appropriate referral for affected individuals in sub-Saharan Africa. This will be achieved by: (i) Building an evidence-base through surveillance and research while strengthening service capacity; (ii) Improving collaboration across research, care and advocacy groups; and (iii) Actively engaging to promote policies that supports these aims.

The **objectives** are:

1. Provision of a collaborative research and practice forum for Network members.

2. Sharing, analysis and dissemination of harmonized surveillance data regarding the prevalence and prevention of congenital anomalies, and care of affected individuals.

3. Establishment of platforms that support cross-cutting stakeholder engagement to create effective impact pathways at local, national and regional levels.

4. Support for capacity-building relevant to diagnosis, surveillance, research, prevention, and care through training and other resources.

5. Raise awareness and address barriers and stigma by developing and/or disseminating appropriate public communication materials.

6. Share best practice and innovative approaches to local challenges using technology and other resources.

These will be operationalized through:

1. The maintenance of the sSCAN website (https://sscan.tghn.org/) with links to sSCAN events and resources; and other congenital anomaly-related resources.
2. Regular webinars conducted by sub-Saharan African experts (regional research, clinical practice, public health interventions and patient support).
3. Support for networking forums (on-line and in-person) and meetings for engagement and support.
4. Development of data transfer standards and protocols to support data-sharing.
5. Position statements/publications – policy advocacy.

Membership

Eligibility: Any Organization or Person active in congenital anomaly surveillance, research, or care in sub-Saharan Africa, whether epidemiological, clinical, social or biomedical.

There will be three types of membership:

1. *A "Member Organisation" (Full Member) will join as a surveillance or research programme, a department (e.g., of a University), organisation or institution based in sub-Saharan Africa. There will be a lead contact name associated with the organisation who will be authorised to represent it at sSCAN. The Organisation will be described on the sSCAN website and is authorised to reflect the sSCAN affiliation on its website. The Organisation will be expected to participate in relevant collaborative studies or data sharing activities (as appropriate), to attend annual/biennial meetings, and to participate in one of the sSCAN project/administrative sub-committees. When funding is available, it may be used to cover attendance of up to three members of the Organisation*

to meetings, but otherwise organisations will be expected to fund meeting attendance themselves. Funding priority will be given to partners based in sub-Saharan Africa. Member Organisations may include congenital anomaly surveillance programs, clinical departments, public health departments, patient groups, professional associations, and others. As Full Members, Member Organisations will be afforded voting rights in selection of the sSCAN Director and Steering Committee (three votes per Organization). Organisation Membership will be approved by the Steering Committee.

2. *A "Member Individual" (Full Member) can join as an individual, whether or not they are also part of a Member Organisation. The Individual's work should be related to diagnosis, care, and prevention of congenital anomalies. They should be a national of a sub-Saharan Africa country with their primary research focus based in sub-Saharan Africa. The Individual will be listed on the sSCAN website. The Individual will be expected to attend annual/biennual meetings, and to participate in at least one sSCAN sub-committee. They will be invited to participate in collaborations where appropriate. Their attendance at in-person meetings may be supported if funding is available, but otherwise they will pay for this themselves. Individual members may be researchers, academics, clinicians (including medical, nursing, rehabilitation), public health professionals, patient representatives. As Full Members, Member Individuals will be afforded voting rights (one vote per Individual) in selection of the sSCAN Director and Steering Committee, provided that they do not belong to a Member Organization with voting rights. Individual Membership will be approved by the Steering Committee.*

3. *A "World Affiliate" is an interested person or organisation based outside sub-Saharan Africa who wishes to be kept informed of sSCAN activities and attend meetings (at their own expense). An Affiliate's work should be related to diagnosis and prevention of congenital anomalies and/or care for affected individuals in sub-Saharan Africa. Affiliate Members will not have voting rights in selection of the sSCAN Director and Steering Committee. Affiliate Members can include trainees and other international colleagues. Affiliate Membership will be approved by the Steering Committee.*

A membership fee will be used to maintain the website (supported by The Global Health Network) and minimal administrative functions. All fees are presented in US dollars. The subscription period is one year and there are no automatically recurring payments. It is suggested that Members include annual fees in their grant applications. Motivations to waive fees will be reviewed by the Steering Committee. Member Organisation subscription will be dependent on a membership category based on location, size and annual budget of the organisation. Member Individual subscription will be dependent on membership category based on location i.e. low, middle or high-income country.

The benefits of Full Membership include: the opportunity for sub-committee and Steering Committee membership and leadership positions; data-sharing and access to pooled data for approved concepts; using the platform for networking and collaboration with partners.

World Affiliate Members will benefit from close liaison with sSCAN. They can participate in meetings and specific projects.

Organisational Structure

The organizational structure of sSCAN includes the following bodies described below (Figure 1):

General Assembly comprising sSCAN membership

Director elected/approved by the Steering Committee will be supported by an Administrative Team

Steering Committee elected/approved by the General Assembly and including the Chairs of the Sub-Committees

Portfolio Committees

Project Sub-Committees

International Advisory Committee

The sSCAN General Assembly is the final decision-making body of the Network; it will be responsible for the main strategic decisions and the long-term direction. The General Assembly will be held once a year/every two years and comprises all Full Members.

The sSCAN Steering Committee is the supervisory body for the execution of the activities of the Network; and will be responsible for overseeing the coordination of tasks and compliance within the set deadlines, will review membership applications and report the actions taken to the General Assembly. Members of the Steering Committee will agree on data requests from members and discuss and approve funding applications involving sSCAN. The Steering Committee members will be elected every two years by voting in the General Assembly. The Steering Committee will be chosen from members and will be composed of five members (in addition to the Director and a representative from each sub-committee) Membership of the Steering Committee should be diverse representing **different sub-Saharan African countries** and **different disciplines**. The members of the Steering Committee should be based at an organization in sub-Saharan Africa, resident in sub-Saharan Africa or have their main research focus based in sub-Saharan Africa.

The term of a Steering Committee membership will be two years, with an option to extend two further years. The Steering Committee will meet virtually minimum of three times per year, and in person at the time of the General Assembly meeting.

Figure 1. sSCAN Organogram

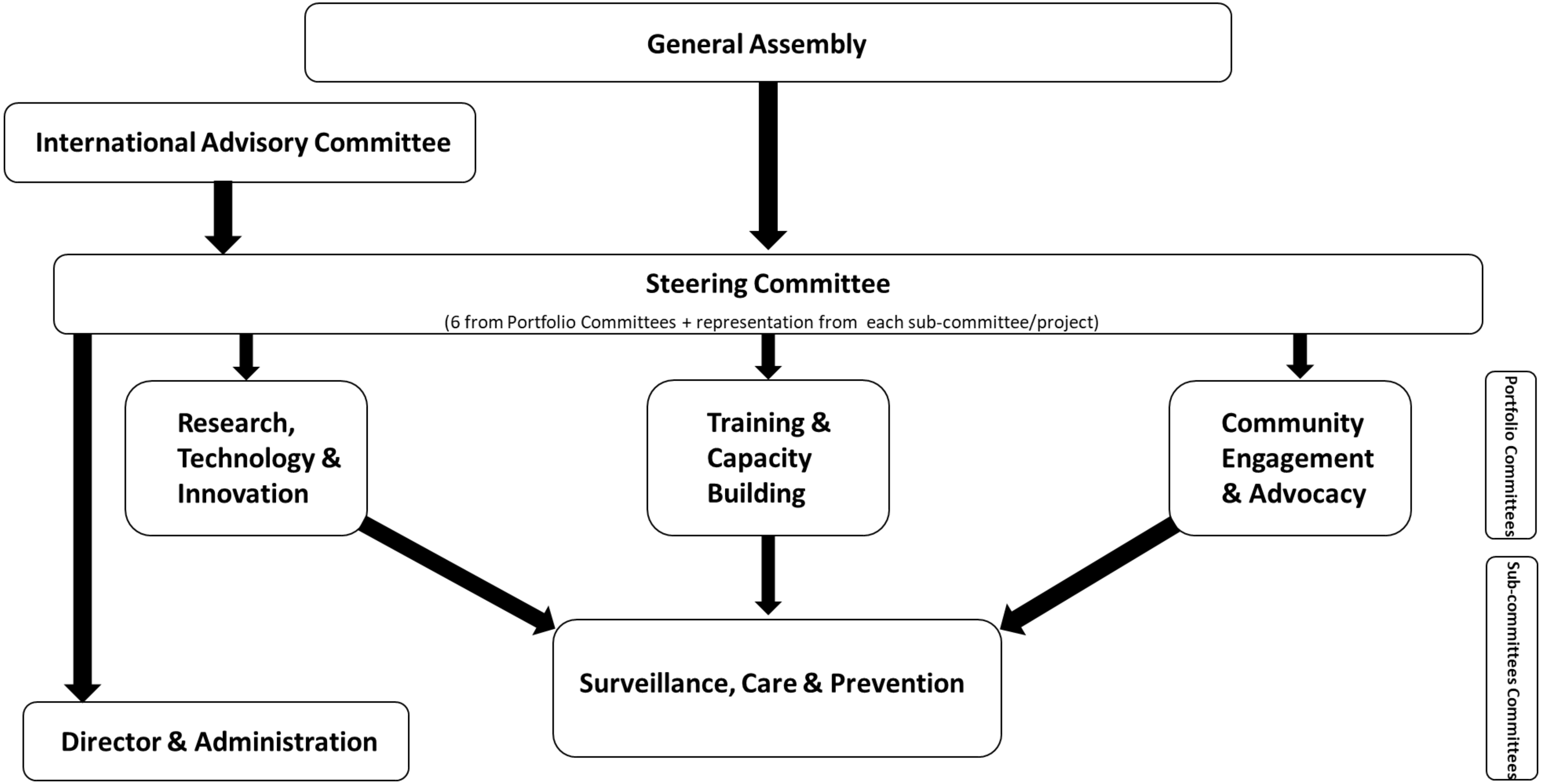

The Director is responsible for hosting sSCAN infrastructure and managing administrative processes. This includes hosting the joint databases, sourcing funding for the network with the support of the Steering Committee, and managing grant contracts. The Director and will report to the Steering Committee and General Assembly. Administrative support will be based at the institution of the Director.

The Steering Committee will issue a call for applications for Director for a period of not less than five years, and applications will be assessed by the Steering Committee (by voting if necessary) and validated by the General Assembly. The Director must be resident in sub-Saharan Africa. If after five years, both Director and Steering Committee agree, the Directorship can extend for a further five-year term, with a limit of two consecutive terms served. If the Steering Committee wish, they can issue a new call for Director applications at the end of any five-year term. If the Director resigns during a five-year term, a call will be made for applications for a new Director, who will work with the existing secretariat until the end of the term. The Director will be expected to contribute a minimum of 20% of their time, free of charge if no funding contract exists, to the running of the Network.

Most activities are inter-connected (research-technology-patient care-advocacy) and categories are artificial. However, for ease of operations, the Steering Committee will have three broad Portfolios: Research, Training & Capacity Building, and Community Engagement and Advocacy. Network and individual projects/sub-committees can fit into and across these portfolios and include each of them as aims within the individual study. Each Portfolio Committee should have a Chair/Champion who manages the groups and keeps members accountable for activities and outputs. Portfolio Committees will be decided by the General Assembly and will be long-term committees dedicated to specific broad activities. Each Portfolio Committee shall elect its own Chair who must be a full member of sSCAN (either Individual Member or belonging to a Member Organization). The Portfolio Chair will serve for a two-year term with the option to extend for a further two years with a limit of two consecutive terms. Portfolio Committees can include World Affiliates. The Chair of each Portfolio Committee will report to the Steering Committee (or appoint a rapporteur) on projects, outcomes and dissemination of information relevant to the Portfolio.

Sub-committees/Project Committees will fall within the Portfolios and be established gradually as the Network builds capacity. Each sub-committee shall elect its own Chair who must be a full member of sSCAN (either Individual Member or belonging to a Member Organization). The sub-Committee Chair will serve for a two-year term with the option to extend for a further two years with a limit of two consecutive terms. Sub-Committees can include World Affiliates. The Chair of each sub-Committee will report to the Steering Committee (or appoint a rapporteur) on projects, outcomes and dissemination of information relevant to the sub-Committee.

International Advisory Committee (IAC)

The sSCAN Steering Committee will invite members to join an International Advisory Committee (IAC) with its own Terms of Reference. The sSCAN IAC will include representatives of organisations or individuals with experience relevant to the activities of sSCAN, who will give scientific, policy, operational or advocacy advice, and assist with circulating the activities of sSCAN globally. The sSCAN IAC will meet virtually at least once a year, with a Chair who will serve a term of two years (See sSCAN International Advisory Committee Terms of Reference).

Data Sharing

Sharing data regionally is critical to the mandate of sSCAN. The types of data shared (aggregate data, individual depersonalized data, data on health services, resources and use) and methods of sharing will be decided by members on a per project basis according to the capabilities of each organisation and the legal and ethical environment of the institutions and countries involved.

Terms and conditions relating to data sharing will be signed by each Member Organization.

Data will remain under the ownership of the contributing Member Organization, whose formal permission must be obtained for *each* publication, whether on the sSCAN website or in journals or reports. Secure storage, analysis and dissemination of results will be according to best international practice, as agreed by the Steering Committee and in line with the data protection regulations on each contributing country and institutions and the country and institution hosting the Directorate.

Authorship of collaborative publications will be decided according to the guidelines of the International Journal of Medical Editors (IJME), but it is expected that data contributors would ordinarily meet these criteria and be co-authors.

- Substantial contributions to the conception or design of the work; or the acquisition, analysis, or interpretation of data.
- Writing the work or critical review of important intellectual content; and
- Final approval of the version to be published; and
- Agreement to be responsible for all aspects of the work, ensuring that issues related to the accuracy or integrity of any part of the work are properly investigated and resolved.

Transition from Seed Project to the new Governance Structure

The first hybrid sSCAN General Assembly was held in Kampala, Uganda in March 2022. There was extensive discussion of the draft governance documents, the summary of which is presented here.

Linda Barlow-Mosha was the acting- Director until the end of the Seed Project. In the absence of formal application process to the Steering Committee, engaged co-investigators comprised the initial Steering Committee which maintained the sSCAN website and webinar programme, prepare conference presentations, initiated funding applications and organize the second sSCAN annual meeting. A formal process to accept the Governance Structures and appoint a Steering Committee for the next two years (2023/2025) will be conducted at the second sSCAN General Assembly 11 – 13 September 2023 in Cape Town, South Africa.
